# Novel method of boundary-free mesh parameterization

**DOI:** 10.1371/journal.pone.0217537

**Published:** 2019-06-06

**Authors:** Liming Duan, Xueqing Luo, Lang Ruan, Minghui Gu

**Affiliations:** 1 ICT Research Center, Key Laboratory of Optoelectronic Technology and System of the Education Ministry of China, Chongqing University, Chongqing, China; 2 College of Mechanical Engineering, Chongqing University, Chongqing, China; 3 Engineering Research Center of Industrial Computed Tomography Nondestructive Testing of the Education Ministry of China, Chongqing University, Chongqing, China; University of New South Wales, AUSTRALIA

## Abstract

Unless the targeted mesh is developable, metric distortion is inevitable during the process of surface mesh parameterization, thus one important objective of all involved parametric studies is to reduce the metric distortion. In order to further reduce area and angle distortion, a novel method of boundary-free mesh parameterization is presented in the paper. Firstly, the initial boundary-fixed conformal parameterization from 3D surface mesh patch to a plane is performed in the method. Then, based on the initial parameterization, the iterations of boundary-free quasi-harmonic parameterization are developed, where the tensor field is updated in each iterative step and the principal curvature direction is utilized to terminate the iteration. The solution of the novel method is convenient to calculate since it involves a series of linear systems. In our novel parameterization method, lower metric distortion and considerable efficiency have been obtained in experiments.

## Introduction

3D surface mesh parameterization is an important topic in in mechanical engineering research. Many computer-aided applications (such as mesh processing in reverse engineering, mesh generation of finite element analysis and finite element simulation) that are commonly used are based on mesh parameterization. Therefore, with the development of the computer industry, It is more and more important to find a good parameterization method. [1].

Mesh parameterization involves computing the mapping between a triangulated mesh surface and certain parametric domain. It is well known that, unless the targeted surface mesh is developable, mesh parameterization inevitably incurs some metric distortions in both angle and area. Therefore, to find the parameterization which preserves the geometric properties of original 3D mesh as much as possible, that is, to try to reduce angle and area distortion, it has been the major aim of mesh parameterization since its appearance. There are two types of mesh parameterization goals: authalic (area-preserving) mapping and conformal (angle-preserving) mapping. The construction of conformal (angle-preserving) parameterization is relatively easy to solve. In fact, the construction of authalic (area-preserving) parameterization which is general to reduce area and angle distortion is still a challenging problem and the previous involved methods are all non-linear calculations. Compared with non-linear ones, linear methods are preferred in parametric studies for the reason that they have shorter running time and smaller implementation complexity.

Surface mesh parameterization emerged in early years of 1990s. Detailed reviews of mesh parameterization are referred in the relevant comprehensive references[2,3,4,5]. We concerned merely the most relevant work of mesh parameterization in the paper. In mesh parameterization, the set boundary shape of the parametric domain has a significant influence on metric distortion. It is crucial to take the boundary shape into account in the setup process of parameterization. There are two types of the mesh parameterization methods: boundary-fixed ones and boundary-free ones. Compared with that with constrained boundary, mesh parameterization with free boundary could achieve better results for its lower metric distortion. In this paper, we consider boundary-free parameterization to parameterize a surface mesh patch with a disk topology onto a plane without constrained boundary.

In boundary-fixed parameterization, the angle and area distortion is considerable due to constraint of fixed boundary. Furthermore, boundary-fixed parameterization is far away from isometric for reason that it’s merely guaranteed to be as conformal as possible. To further reduce metric distortion and improve parameterization, the most straightforward method is to release boundary constraints of parametric domain. At present, the fixed boundary parameterization method of spherical parameterization has obtained better effect. Chao Peng et al. introduced an efficient method to establish surface correspondences between genus-zero triangle meshes and animate a morph between them[6]. Xin Hu et al. presentd a practically robust approach to compute high-quality spherical parameterizations with bijection and low isometric distortion[7]. Choi et al. proposed a fast algorithm to compute the optimized spherical harmonic parameterization with consistent landmark alignment[8], also, they proposeed an iterative scheme called the north-south reiteration for achieving a spherical conformal parameterization[9]. Although they had addressed similar issues as those presented in our method in distortion reduction, because they were not a very suitable parametric domain for surface models, they were not able to get the ideal parameterized result. Through the constructive definition of the general convex space of piecewise linear mapping, Lipman guaranteed the largest conformal distortion and the local and global injection of their mappings. This method showed how common geometric processing objective functions can be restricted to these new spaces, rather than the entire spaces of piecewise linear mapping, to provide a bounded version of the popular algorithm[10]. In [11], Aigerman and Lipman developed an algorithm for computing bounded distortion mapping in 3D. The algorithm can be applied for parameterizing meshes to the 2D planes or polyhedrons. By growing “virtual boundary” which absorbed the distortion partly induced by the boundary, Lee et al. embedded the surface mesh patch into an increasing larger planar patch[12]. In addition, a non-linear method was presented, which combined virtual boundaries with scaffolding triangles to achieve parameterization with partial free-boundary[13]. Besides, other certain parameterization methods were presented whose boundary conditions only required fixing (at least) two boundary vertices[14,15].

Boundary-free parameterization is superior to boundary-fixed parameterization on both area-preserving and angle-preserving, since it runs without any constraints on the boundary. The most prominent method of boundary-free parameterization is the angle based flattening (ABF) which formulated the problem as a constrained non-linear optimization of angles[16]. ABF generates conformal mappings with great computational complexity, and it has been further developed, e.g. its efficient and robust optimization: ABF++[17]. In spite of the improved efficiency, the calculation of ABF++ is same as non-linear of ABF, hence later a complete reconstruction of ABF emerged which possessed the linear solution marked as LABF[18]. Liu et al. proposed a local/global linear boundary-free method, which was different from conventional ones to map the entire vertices on the surface mesh patch on plane, and it parameterized all the triangles on a surface mesh patch onto a plane and tried to force each 2D triangle of the parameterized patch to be an version of its 3D counterpart as similar as possible[19]. By finding the largest eigenvalue/eigenvector of a sparse symmetric matrix, Mullen et al. presented a spectral method to obtain automatically boundary-free conformal parameterization of surface mesh patch, where high-quality parameterized results were achieved [20]. With the help of the distance from a centre vertex to all the boundary vertices, Jun-jie C. et al. proposed a simple and fast method of measured boundary-free parameterization to achieve the goal of minimizing the conformal distortion[21].Lam et al. presented a variational algorithm to compute the optimized quasi-conformal parameterization with controllable area distortions by controlling the Beltrami coefficient to guarantee the conformality of the parameterization[22].The quasi-conformal parameterization (QCMC) is one of iterative algorithms, which can simultaneously search for the conformal module and the optimal quasi-conformal parameterization by minimizing the Beltrami energy with the conformal module of the parameter domain incorporated[23].T-Map is the latest and most effective way to compute conformal parameterization. An efficient iterative algorithm (QCTM), called the quasi-conformal iteration, was proposed to compute a unique T-Map between two surfaces which minimizes the maximal conformality distortion[24,25]. For the same purpose, another iterative algorithm was proposed to compute the extremal T-Map using the Beltrami holomorphic flow (BHF) which produces a sequence of quasi-conformal mappings converging to the T-Map to minimize the conformality distortion[26].

Listed above boundary-free parameterization methods are targeted to be as conformal as possible, whose angle-preserving effects are obviously better than boundary-fixed ones. But they didn’t consider the authalic issue, and their effects of area-preserving are dissatisfied in general. Discrete tensorial quasi-harmonic parameterization premeditates area-preserving on basis of conformal issue, which is a significant kind of method resulted in both angle-preserving and area-preserving performance[27,28]. It relies on the linear operator to capture parameter distortion in the form of local deformation tensors which are used as guiding fields in a manner similar to the Poisson equation setting. In fact, it tries to reduce the distortion of an initial boundary-fixed parameterization by further computing a plane-to-plane parameterization which reproduces a Jacobian as close to the Jacobian of initial parameterization as possible.

Inspired by applications in mesh parameterization, Claici et al. presented a new preconditioning technique for large-scale geometric optimization problems [29]. Kovalsky et al. presented the Accelerated Quadratic Proxy (AQP)—a simple first order algorithm for optimizing geometric energies defined over triangular and tetrahedral meshes[30].These two methods have a great contribution in accelerating the parameterization process. Inspired by these methods, we have added a method of energy minimization to the method in this paper, the solution of the novel method involves a series of linear systems, which facilitate calculation and improve efficiency.

Dong et al. developed a iterative method to compute a boundary-free quasi-conformal parameterization, which fitted the parameter coordinate gradients to two orthogonal guiding vector fields with equal magnitude[31]. Motivated by the methods in [28] and [31], a novel boundary-free parameterization method to parameterize a surface mesh patch with a disk topology onto plane is presented in the paper which may be considered as an improved version of parameterization methods in [28] and [31]. Our method whose key-point was iterative, substantially the same as the method in [28], was to find a most isometric parameterization, and we took the method from [28] and extended it with the guiding fields idea from [32]. Compared with previous boundary-free parameterization methods, our method could obtain a considerable efficiency and achieve more isometric results which have lower area and angle distortion by running the iterations which set the different local correctional tensors in each iterative step. n

The remainder of the paper is organized as follows. Section 2 states the involved background knowledge. Section 3 describes the novel method. Section 4 introduces setting of differential operators of our method. Section 5 elaborates our method in detail. Section 6 outlines the experiments and the discussion about several mesh models. The paper is concluded in section7.

## Background knowledge

A parameterization is usually generated with boundary restrictions. Given appropriate Dirichlet condition on the boundary, the resulting parameterization is guaranteed to be a one-to-one mapping. For example, as the parameterized domain is restricted within a planar unit circle, the Dirichlet condition on the boundary is

$$u^{2}+\nu^{2}=1.$$

When computing the parameterization, the system of solution must consist of the regular solving system and a restricted equation

$$u^{2}+\nu^{2}\leq 1.$$

However, the novel parameterization method in the paper is a free boundary, so there is no need to consider Dirichlet boundary condition when computing the parameterization.

The goal of the paper is to calculate the boundary-free parameterization which maintains angle-preserving and area-preserving (i.e., isometric) as much as possible. Relying on certain knowledge of metric distortion, conformal parameterization and quasi-harmonic parameterization, our parameterization method was developed

### Conception of metric distortion in parameterization

To better understand metric distortion in parameterization, let us see what happens to the surface point *f*(*u*,*v*) as we move a tiny little away from (*u*, *v*) in the parameter domain. If we denote this infinitesimal parameter displacement by(Δ*u*,Δ*v*), then the new surface point *f*(Δ*u*,Δ*v*) is approximately given by the first order Taylor expansion *f* of *f* around (*u*,*v*),

$$\tilde{f}(u+\Delta u,\nu +\Delta \nu )=f(u,\nu )+f_{u}(u,\nu )\Delta u+f_{v}(u,v)\Delta v.$$

**Fig 1:**
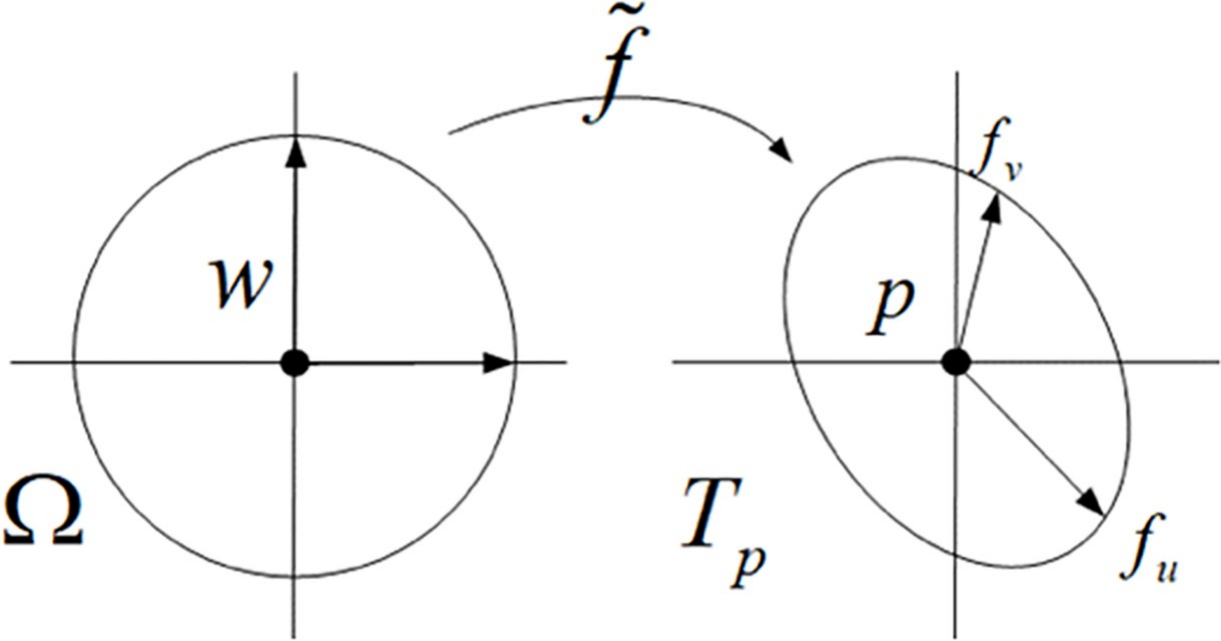
First order Taylor expansion $\tilde{f}$ of the parameterization *f*.

This linear function maps all 3D-surface points in the vicinity of *w* = (*u*,*v*) into the tangent plane T_*p*_ at 3D-surface point *p* = *f*(*u*,*v*)∈*S* and transforms circles around *w* into ellipses around *p* (Fig 1). The latter property becomes obvious if we make the Taylor expansion more compact as

$$\tilde{f}(u+\Delta u,v+\Delta v)=p+J_{f}(s)\left(\begin{array}{l} \Delta u\\ \Delta v \end{array}\right),$$

where *J*_*f*_ = (*f*_*u*_ *f*_*v*_) is the Jacobian of function *f*, i.e. the 3×2 matrix with the partial derivatives of *f* as column vectors. Then using the singular value decomposition of the Jacobian,

$$J_{f}=U\Sigma V^{T}=U\left(\begin{array}{cc} \sigma_{1} & 0\\ 0 & \sigma_{2}\\ 0 & 0 \end{array}\right)V^{T},$$

with singular values *σ*_1_≥*σ*_2_>0, orthonormal matrices *U*∈*R*^3×3^ and *V*∈*R*^2×2^ with column vectors *U*_1_, *U*_2_,*U*_3_, and *V*_1_,*V*_2_ respectively.

The transformation of circles into ellipses is called metric distortion of the parameterization as it shows how *f* behaves locally around some parameter point *w*∈*Ω* and the corresponding surface point *p* = *f*(*w*)∈*S*. All information about the metric distortion is hidden in the singular values *σ*_1_ and *σ*_2_. If both values are identical, then *J*_*f*_ is just a rotation plus uniform scaling and *f* does not distort angles around *w*, we say that *f* is angle-preserving, i.e. conformal; if the product of the singular values is 1, the area of any circle in the parameter domain is identical to the area of the corresponding ellipse in the tangent plane and we say that *f* is locally area-preserving, i.e. authalic.

We now summarize the main properties that the parameterization can have locally:

*f* is isometric or length-preserving ⇔ *σ*_1_ = *σ*_2_ = 1,

*f* is conformal or angle-preserving ⇔ *σ*_1_ = *σ*_2_,

*f* is authalic or area-preserving ⇔ *σ*_1_*σ*_2_ = 1.

Obviously, any isometric mapping is conformal and authalic, and every mapping that is conformal and authalic is also isometric, in short,

Isometric ⇔ conformal + authalic.

In the process of obtaining the optimal parameter, it is necessary to construct an energy function to measure the size of the parameterized metric distortion, and minimize the energy functional. This idea is similar to [33].The most common and classical energy function in mesh patch parameterization is the Dirichlet function[34]:

$$E_{D}=\frac{1}{2}\int_{M}\|\nabla u{\|}^{2}+\|\nabla v{\|}^{2}.$$

### Knowledge of conformal parameterization

In the past two decades, surface conformal parameterization has been widely studied[35].According to boundary constraint, There are two types of conformal parameterization: boundary-fixed and boundary-free. Boundary-fixed conformal parameterization attains the minimum of the quadratic Dirichlet energy:

$$E_{D}=\frac{1}{2}\int_{M}\|\nabla u{\|}^{2}+\|\nabla v{\|}^{2},$$

whose solution is the root of the Laplace equations[1] (for specific principles, please refer to the literature [32]:

$$\left\{ \begin{array}{l} \Delta u=0\\ \Delta v=0 \end{array}\right..$$

Constrained by the piecewise-linear representation of the solution, boundary-fixed parameterization is far from the actual conformal demand. For more details on constrained mesh parameterization, please refer to the literature [36]. Thus further exploration on boundary-free has been carried on as follows.

It is wished to find an ideal pair of parametric coordinate functions(*u*, *v*):*V*→*R*^*2*^ with the property that

∇u=R∇v, (1)

where the operator *R* denotes a counter-clockwise rotation of 90ºaround the surface outward normal. This is just one representation of the well-known Cauchy-Riemann equation which is the necessary and sufficient condition to be conformal completely on parameterization[31]. For a surface mesh patch, no such piecewise-linear parameterization with complete conformality exists unless it is developable. In practice, thus a parameterization tends to be found which is as conformal as possible, in other word that the parameterized coordinate functions (*u*,*v*) would like to be searched that be as far as possible to meet Cauchy-Riemann Eq (1). Therefore, the following solution for (*u*,*v*) is proceeded.

It is trivial to find two vector fields(*g*_1_,*g*_2_):*F*→*R*^3^, where *g*_2_ = *Rg*_1_. The targeted coordinate functions(*u*,*v*) would like to be found whose gradient fields approximate most closely to the guiding vector fields(*g*_1_,*g*_2_):

$$\underset{\left(u,v\right)}{\min}\int_{M}\|\nabla u‐g_{1}{\|}^{2}+\|\nabla u-g_{2}{\|}^{2}$$ (2)

In effect, it is just the boundary-free conformal parameterization that attains the minimum of the quadratic Dirichlet energy without boundary conditions:

$$E_{D}=\frac{1}{2}\int_{M}\|\nabla u‐g_{1}{\|}^{2}+\|\nabla \nu -g_{2}{\|}^{2}.$$

It is well known that the optimization variational problem formed as formula (2) may be solved by Poisson equations:

$$\left\{ \begin{array}{l} \Delta u=divg_{1}\\ \Delta \nu =divg_{2} \end{array}\right.,$$ (3)

where *g*_1_ and *g*_2_ are a pair of guiding vector fields[18].

### Knowledge of quasi-harmonic parameterization

Quasi-harmonic parameterization was claimed to find a most isometric map. In fact, it is a map from a plane to another plane, and proceeds as a further procedure after initial conformal parameterization from 3D mesh to the plane.

Discrete tensorial quasi-harmonic parameterization attempts to reduce the metric distortion by minimizing the quasi-harmonic energy:

$$E_{\mathrm{OH}}=\int_{M}(C\nabla u)\;\nabla u+(C\nabla v)\;\nabla v,$$

where the tensor field *C* is determined as follow: Given an initial convex boundary-fixed conformal parameterization, such a 2×2 tensor

$$C_{j}={\left(J_{j}^{T}J_{j}\right)}^{\frac{1}{2}}$$

of per triangle *j* in *M* is defined, where *J*_*i*_ denotes the 3×2 Jacobian of the initial conformal parameterization.

The partial differential solution associated with minimizing the quasi-harmonic energy *E*_*OH*_ is an alignment quasi-harmonic equations:

$$\left\{ \begin{array}{l} div(Cgradu)=0\\ div(Cgradv)=0 \end{array}\right..$$

Quasi-harmonic parameterization establishes plane-to-plane map which mimic the original 3D-mesh shape not only in angle but also in area for the reason that *C* captures the properties of Jacobian of the initial boundary-fixed conformal parameterization.

Building upon the Poisson setting in boundary-free conformal parameterization, quasi-harmonic parameterization is easily extended to boundary-free case which is achieved by applying its variant:

$$\left\{ \begin{array}{l} div(Cgradu)=divg_{1}\\ div(Cgradv)=divg_{2} \end{array}\right.,$$

where *g*_1_ and *g*_2_ are the pair of guiding vector fields.

## Description of novel method

Our novel boundary-free parameterization method would result in an isomorphic planar mesh *U* (whose vertices set has vertices each with 2D coordinates(*u*,*v*)) mapped from a given surface mesh patch M(whose vertices set *V* has vertices each with 3D coordinates(*x*,*y*,*z*)) with a disk topology.

**Fig 2:**
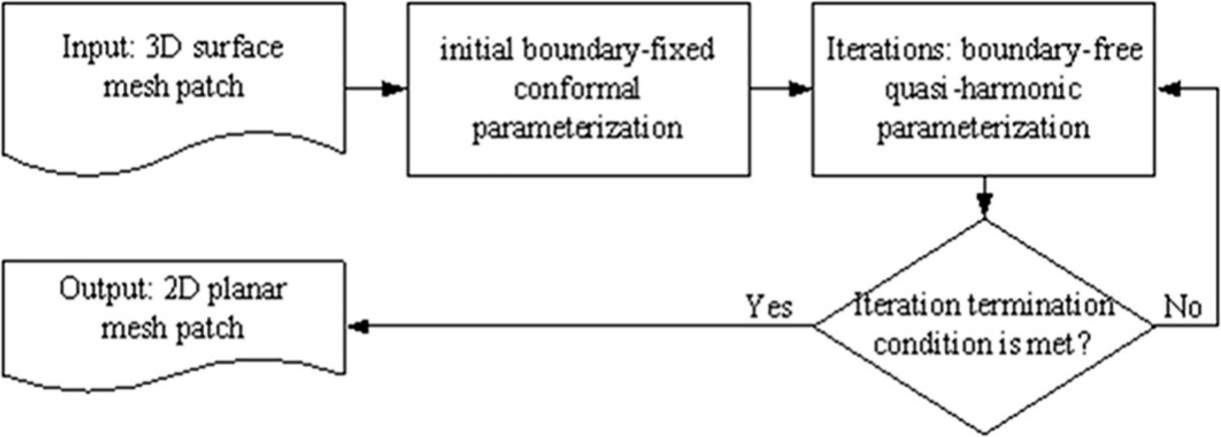
Novel method flow.

Our method consists of two steps: initial boundary-fixed conformal parameterization and boundary-free quasi-harmonic parameterization. Our method flow is illustrated in (Fig 2).

The first step, initial boundary-fixed conformal parameterization, is expressed in form of

$$\left\{ \begin{array}{l} div(\nabla u)=\Delta u=0\\ div(\nabla \nu )=\Delta \nu =0 \end{array}\right.$$

as described in section 2, which is the base and pre-step of our novel method. In above formulas, the unknowns are the result parameterized coordinates (*u*,*v*) of all mesh vertices on plane *Ω*; the known items are the 3D coordinates (*x*,*y*,*z*) of all mesh vertices on the original surface *S*. A series of linear equations corresponding to all mesh vertices for *u* and *v* could be solved, thus obtaining parameterized result coordinates (u, v) for all vertices.

The later step, boundary-free quasi-harmonic parameterization, is iterative and the key to our novelty. It is expressed in form of

$$\left\{ \begin{array}{l} div(C\nabla u)=divg_{1}\\ div(C\nabla \nu )=divg_{2} \end{array}\right.,$$

where *g*_1_ and *g*_2_ are the pair of guiding vector fields, *C* is the sense as explained in section 2.3. Similar as initial boundary-fixed conformal parameterization, the solution process of boundary-free quasi-harmonic parameterization is also to solve a series of linear equations corresponding mesh vertices.

## Setting of differential operators

In conformal parameterization, the differential operator is *div*(*gradu*) and *div*(*gradv*) (namely Δ*u* and Δ*v*), while in quasi-harmonic parameterization, the differential operator is *div*(*Cgradu*) and *div*(*Cgradv*). We take *div*(*gradu*) and *div*(*Cgradv*) as examples to clarify issues related to later content. The 2×2 tensor *C* is piecewise-constant in the surface mesh patch which captures the properties of the Jacobian of the initial boundary-fixed parameterization, and it is constant in each triangle *j* of the mesh remarked as

$$C_{j}={\left(J_{j}^{T}J_{j}\right)}^{\frac{1}{2}}.$$

**Fig 3:**
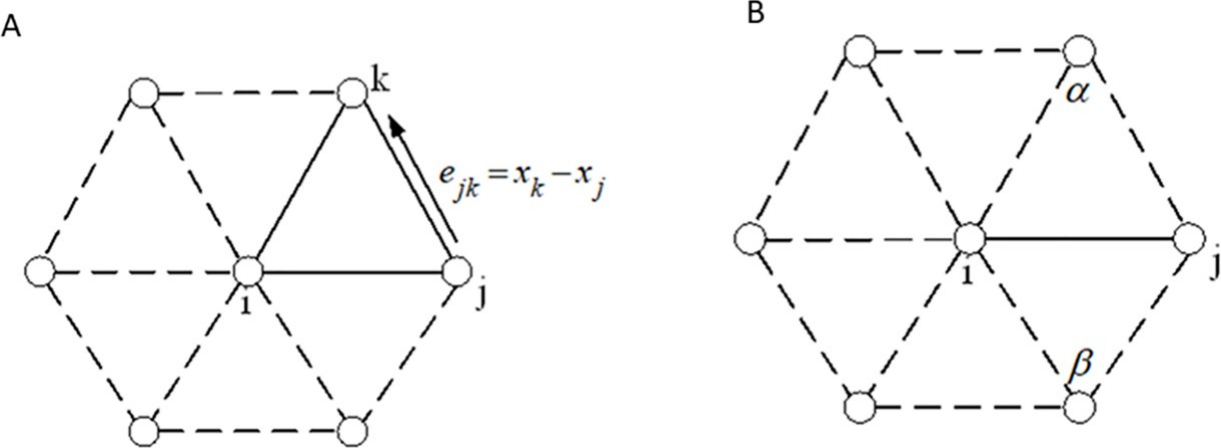
Edges & angles in link of vertex *i*. (A) Edges in link of vertex *i* (B*)* Angles in link of vertex *i*.

On the right side of system (3), the divergence of the vector field g(i.e., *g*_1_ or *g*_2_)at a vertex *i*∈*V* is given as:

divg2=∑(j,k)∈Lkig⋅Rejk,

here *e*_*ik*_ is a edge vector of the mesh and *Lk*_*i*_ is the link of vertex *i*—the set of all edges connected to vertex *i*, as shown in (Fig 3A). The Laplace operator Δ*u* of vertex *i* on surface mesh patch is discretized as a linear system formed as:

$$div_{i}=\Delta_{i}u=\sum_{j\in N_{i}}w_{ij}(u_{i}-u_{j})$$

where *w*_*ij*_ = 0.5×(cot*α*+cot*β*), *N*_*j*_ is the set of all vertices connected to *i*, and *α*,*β* are the two opposite angles of edge (*i*,*j*) on the triangle mesh (Fig 3B)[37].

As for the quasi-harmonic differential operator, it is marked as *div*_*i*_ *(Cgradu)* and *div*_*i*_ (*Cgradv*) on vertex *i* of surface mesh patch. It could also be represented as a linear system, formed as:

divi(Cgradu)=∑j∈Njwij'(ui−uj)

(with regard to parameter *u*), where *w*_*ij*_*ʹ* is interpreted as follows.

**Fig 4:**
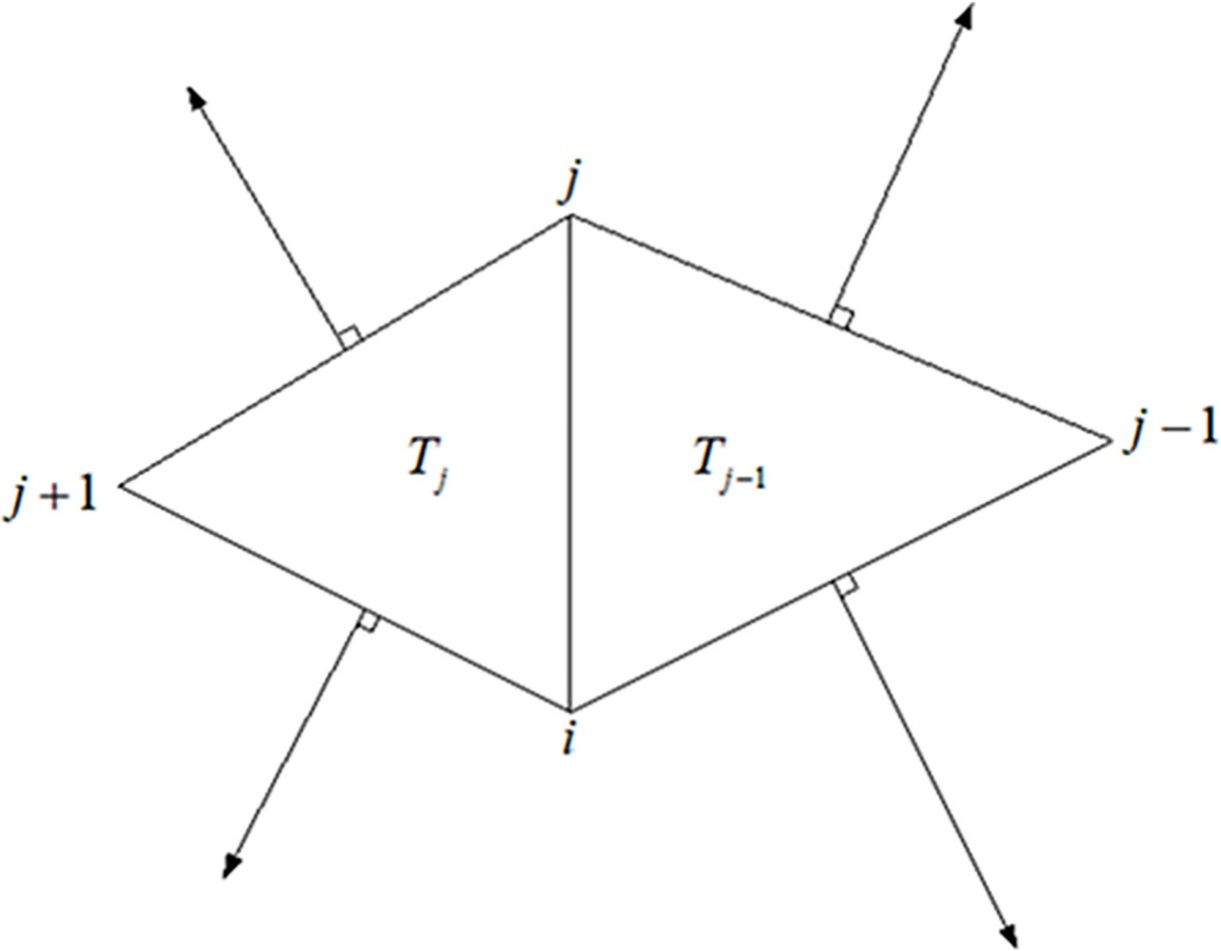
Illustration of coefficients of linear system of quasi-harmonic operator.

Referring to (Fig 4), the coefficient *w*_*ij*_*ʹ* on edge {*i*,*j*} is composed of two half-edge coefficients *w*_*ij*_┴^*j*-1^ʹ and *w*_*ij*_┴^*j*^ʹ, that is, *w*_*ij*_ʹ = *w*_*ij*_┴^*j*-1^ʹ+*w*_*ij*_┴^*j*^ʹ. The half-edge coefficient *w*_*ij*_┴^*j*^ʹ is given as:

wijTj'=xj+1,j⊥⋅(Cjxi,j+1⊥)4Aj,

where *x*┴^*i*,*j*+1^ refers to the vector of planar 2D edge vector *x*^*i*,*j*+1^ rotated by π/2 in its plane and *A*_*j*_ is the area of triangle T_*i*_ = {*i*,*j*,*j+*1}. Thus coefficient *w*_*ij*_ʹ is expressed as

wij'=xj−1,i⊥⋅(Cj−1xj,j−1⊥)4Aj−1+xj+1,j⊥⋅(Cjxi,j+1⊥)4Aj. (4)

## Novel method details

The process of the conformal parameterization method in [31] is iterative. Similarly, the major of our method is iterative, yet the effect of our method is sounder a lot due to involving boundary-free quasi-harmonic parameterization. In addition, by associating the principal directions on vertices of original 3D mesh with termination condition of iteration, the parameterization results of our method would be further maintain area-preserving and angle-preserving. Our method has been proven in experiments, and its effect is superior to other previous methods for its effective improvement brought from the developed iterations.

The two steps, initial boundary-fixed conformal parameterization and boundary-free quasi-harmonic parameterization, are proceed sequentially in our method.

### Initial boundary-fixed conformal parameterization

**Fig 5:**
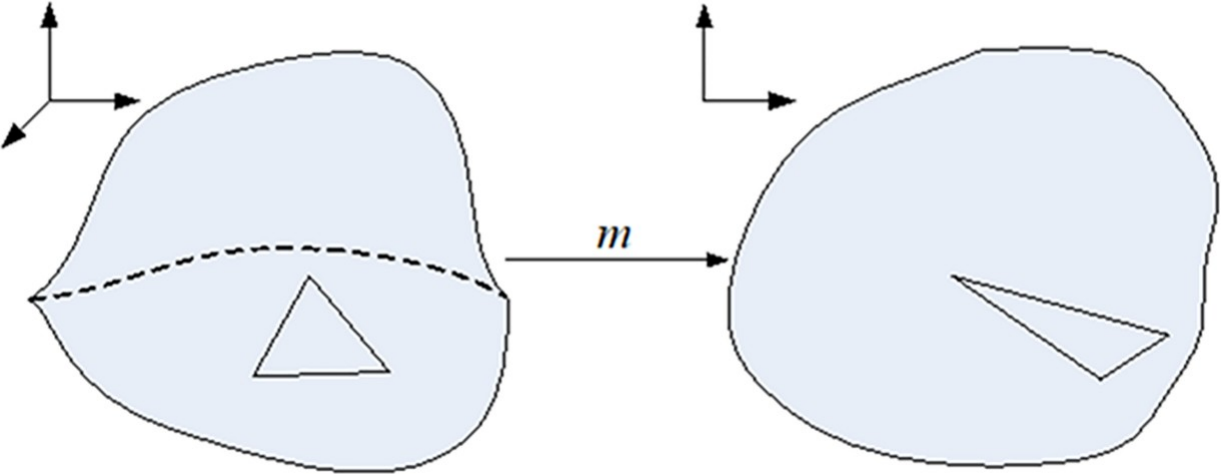
Illustration of the initial boundary-fixed conformal parameterization.

The initial boundary-fixed conformal parameterization is the mapping *m* from 3D surface mesh to plane shown in (Fig 5). On basis of the result parameterization coordinates (*u*,*v*) of the initial parameterization on each vertex of the mesh by solving Laplace equations, further measures could be obtained that the Jacobian *J* of the parameterization of each triangle in the mesh patch:

$$J={\left(\begin{array}{lll} \frac{\partial x}{\partial u} & \frac{\partial y}{\partial u} & \frac{\partial z}{\partial u}\\ \frac{\partial x}{\partial v} & \frac{\partial y}{\partial v} & \frac{\partial z}{\partial v} \end{array}\right)}^{T},$$

the initial gradient fields

$$\left\{ \begin{array}{c} \nabla^{0}u=(\begin{array}{ccc} \frac{\partial u}{\partial x} & \frac{\partial u}{\partial y} & \frac{\partial u}{\partial z}{)}^{T} \end{array}\\ \nabla^{0}v=(\begin{array}{ccc} \frac{\partial v}{\partial x} & \frac{\partial v}{\partial y} & \frac{\partial v}{\partial z}{)}^{T} \end{array} \end{array},\right.$$

and the tensor fields *C*_*i*_ = (*J*_*i*_^T^*J*_*j*_)^1/2^. In the parameterization, the three measures are constant on each triangle of the mesh (i.e., piecewise-constant on the mesh) and would be used in the subsequent procedure of our method.

### Boundary-free quasi-harmonic parameterization

**Fig 6:**
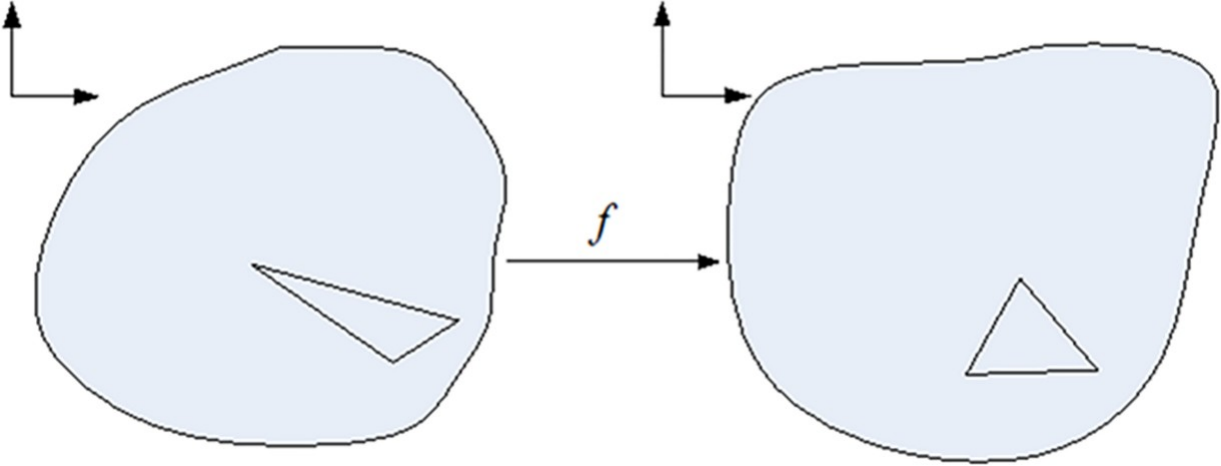
Illustration of boundary-free quasi-harmonic parameterization.

Following the initial boundary-fixed conformal parameterization, the boundary-free quasi-harmonic parameterization, which is in essence the mapping *f* from a planar mesh patch to another shown in (Fig 6), is carried out as the major and innovation part of our method.

The solution of the boundary-free quasi-harmonic parameterization is the system of partial differential equations:

$$\left\{ \begin{array}{l} div(Cgradu)=divg_{1}\\ div(Cgradu)=divg_{2} \end{array}\right.,$$ (5)

where *g*_1_ and *g*_2_ are the pair of guiding vector fields. We would like to improve it into an iterative procedure as our innovation.

**Fig 7:**
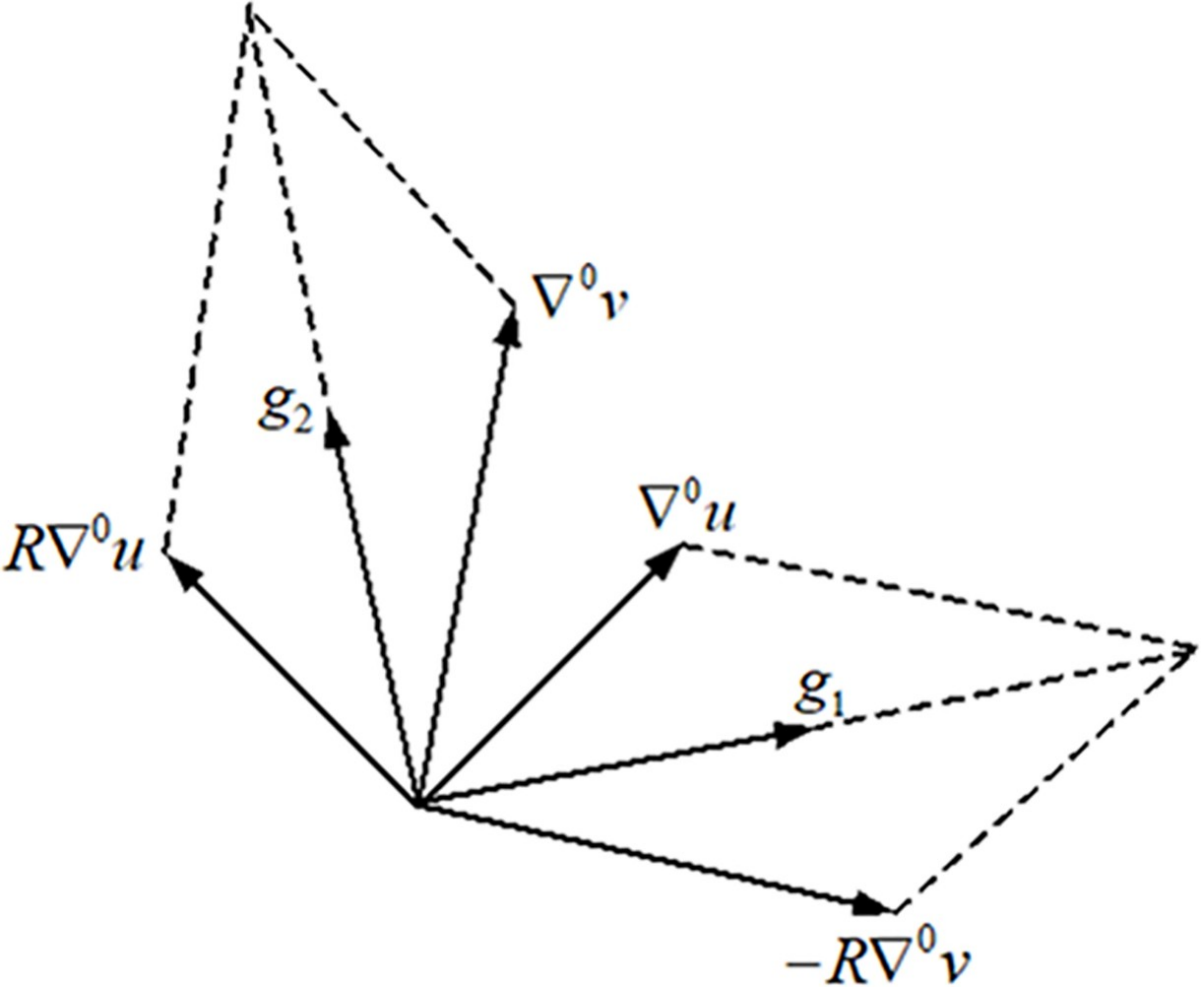
Illustration of constructing two guidance vector fields from two initial vectors.

Firstly same as the boundary-free conformal parameterization in [31], to ensure the parameterization to be as conformal as possible, we would like to find two guiding vector fields to be orthogonal with equal magnitude everywhere: *g*_1_,*g*_2_:*F*→*R*^3^,*g*_2_ = *Rg*_1_. Given the gradient fields ∇^0^_*u*_ and ∇^0^_*v*_ of the initial boundary-fixed conformal parameterization, we could construct the two guiding vector fields *g*_1_,*g*_2_,*g*_2_ = *Rg*_1_, which is expressed by system (6) and illustrated in (Fig 7).

$$\left\{ \begin{array}{l} g_{1}=\frac{1}{2}(\nabla^{0}u-R\nabla^{0}v)\\ g_{2}=\frac{1}{2}(\nabla^{0}u+R\nabla^{0}v) \end{array}\right..$$ (6)

According system (6), the divergences of the two guiding vector fields *g*_1_ and *g*_2_ are obtained as

$$\left\{ \begin{array}{l} div_{i}g_{1}=\frac{1}{2}\left[\nabla^{0}u-\sum_{(j,k)\in Lk_{i}}\left(v_{k}^{0}-v_{j}^{0}\right)\right]\\ div_{i}g_{2}=\frac{1}{2}\left[\nabla^{0}v+\sum_{(j,k)\in Lk_{i}}\left(u_{k}^{0}-u_{j}^{0}\right)\right] \end{array}\right..$$

Thus system (5), the solution of boundary-free quasi-harmonic parameterization, turns out to be

$$\left\{ \begin{array}{l} div_{i}(Cgradu)=\frac{1}{2}\left[div_{i}(gradu)-\sum_{(j,k)\in Lk_{i}}\left(\nu_{k}^{0}-v_{j}^{0}\right)\right]\\ div_{i}(Cgrad\nu )=\frac{1}{2}\left[div_{i}(gradv)+\sum_{(j,k)\in Lk_{i}}\left(u_{k}^{0}-u_{j}^{0}\right)\right] \end{array}\right.,$$ (7)

where the tensor fields *C*_*i*_ = (*J*_*i*_^T^*J*_*j*_)^1/2^ are obtained from the result of initial boundary-fixed conformal parameterization. According to the aforementioned formula:

divi(Cgradu)=∑j∈Niwij'(ui−uj)

and

$$div_{i}(gradu)=\Delta_{i}u=\sum_{j\in N_{i}}w_{ij}(u_{i}-u_{j}),$$

the parameterization results on vertex *i*, i.e(*u*_*i*_,*v*_*i*_). that the coordinates, would be obtained by carrying out system (7). For a surface mesh patch with *n* vertices, this would give two *n×n* sparse linear systems: one for *u* and one for *v*.

Compared with the quasi-harmonic parameterization method in [28],the effect of this one-time boundary-free quasi-harmonic parameterization has been improved a lot. Due to the introduction of the guiding vector fields

$$\left\{ \begin{array}{l} g_{1}=\frac{1}{2}(\nabla^{0}u-R\nabla^{0}v)\\ g_{2}=\frac{1}{2}(\nabla^{0}v+R\nabla^{0}u) \end{array}\right.,$$

which makes the parameterization more conformal, the parameterization results with less angle distortion could be obtained which further correspond to the geometry of original 3D mesh.

### Iterations in novel method

In practice, however the above one-time solution of boundary-free quasi-harmonic parameterization could not meet the required accuracy (i.e., degree of corresponding to the geometry of original 3D mesh) and efficiency (i.e., degree of reducing computational complexity and running time) of the parameterization, thus a new iterative solution was developed to further reduce the metric distortion and improve the efficiency. Inspired by the principle of Seidel iteration in Numerical Analysis, we constructed iterations in our method.

The linear system (7) could be transformed to be an iterative form as system (8) (where the superscript *m* denotes the number of iterations) for achieving better parameterization results with gradually less angle and area distortion along with deepening of the iterations. The tensor field *C* is involved in the iterative process of system (8), and is updated in each step of the iterations, thus obtaining the increased isometric parameterized coordinates (*u*,*v*) step by step. In fact, each iterative step in system (8) plays the same role but with better effect as the later step of the boundary-free conformal parameterization method in [28]. For the timely updated tensor field *C* in the iterations, the better effect of our method is obtained. Due to its many iterative steps, our method has much more running time than the one-time running method in [28], thence it possesses the more effective parameterization results. The solution of iterative system (8) is a series of sparse linear systems, each of which is similar to system (7), linear and convenient to calculate.

$$\left\{ \begin{array}{l} div_{i}(C^{m+1}gradu^{m+1})=\frac{1}{2}\left[div_{i}(gradu^{m})-\sum_{(j,k)\in Lk_{i}}\left(\nu_{k}^{m}-v_{j}^{m}\right)\right]\\ div_{i}(C^{m+1}grad\nu^{m+1})=\frac{1}{2}\left[div_{i}(gradv^{m})+\sum_{(j,k)\in Lk_{i}}\left(u_{k}^{m}-u_{j}^{m}\right)\right] \end{array}\right..$$ (8)

The iterative method of boundary-free parameterization in [31] is just to make parameterization angle-preserving as close as possible. Iterations of the method in [31], each of which fed merely angle distortion of previous iterative step back to current iterative step using Poisson equation, are fundamentally different from ours in the novel method. However, iterations in our method are to feed both angle and area distortion of previous iterative step back to current iterative step, so the boundary-free parameterization in our method is as conformal and authalic as possible. Therefore, our method has less metric distortion in parameterization and the results are more effective than those of the iterative method in [31]. The basic idea of QCTM method[24] is to represent the set of diffeomorphisms using Beltrami coefficients (BCs) and look for an optimal BC associated with the desired T-Map to minimize the maximal conformality distortion. The associated diffeomorphism can be efficiently reconstructed from the optimal BC by using the linear Beltrami solver (LBS). But the iterations of QCTM concern merely also on conformality while not a little on authalic, its authalic effect is better a lot but the conformal effect is just passable.

**Fig 8:**
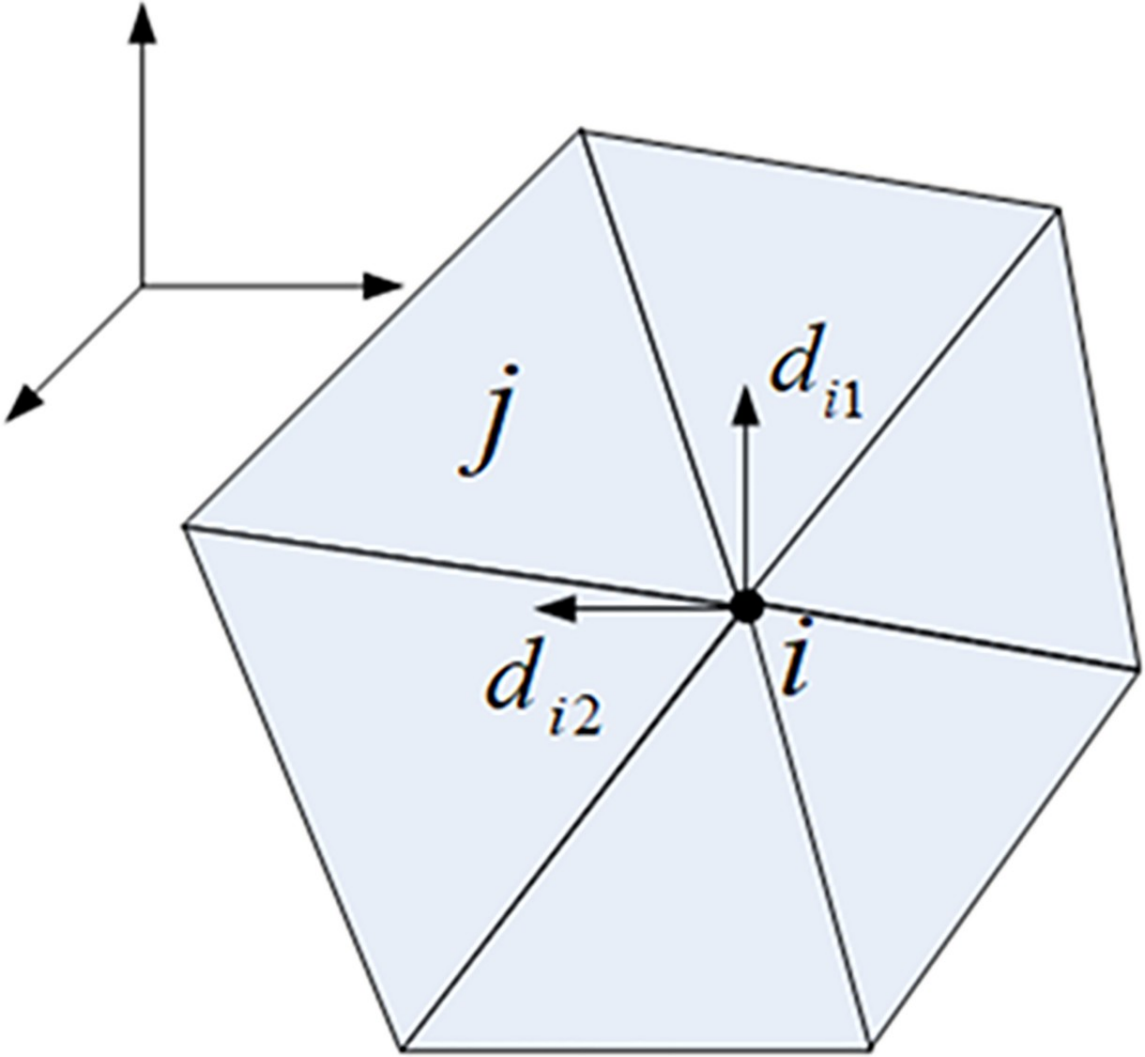
Illustration of parameters in termination criterion *E*_v_.

We designed the termination criterion of our iterations as following. It is well known that the existence of metric distortion in parameterization means that there is a geometrical difference between the result 2D mesh of parameterization and the original 3D mesh. Therefore to minimize the metric distortion of parameterization in our method, we would like to have the parameterized coordinates (*u*,*v*)be more consistent with the geometry of the original 3D mesh when terminating iterations. Thus in certain iterative step, we tried to enable all the resulted coordinate gradients (∇*u*, ∇*v*) on vertices of the mesh to be totally minimally different from the corresponding pairs of the principal directions on vertices. In view of this, we designed a measure of metric distortion as Eq (9), where *N*_*i*_ is the set of triangles possessing vertex *i*, and *d*_*i*1_,*d*_*i*2_ are the two principal directions on vertex *i*, *Area*_*i*_ is the area value of triangle *j*, as illustrated in (Fig 8).

In Eq (9), ∇_*i*_*u* and ∇_*i*_*v* represent parameterized coordinate gradients on triangle *j*; and

$$\frac{\sum_{j\in N_{i}}Area_{j}\nabla_{j}u}{\|\sum_{j\in N_{i}}Area_{j}\nabla_{j}u\|}\;\mathrm{or}\;\frac{\sum_{j\in N_{i}}Area_{j}\nabla_{j}v}{\|\sum_{j\in N_{i}}Area_{j}\nabla_{j}v\|}$$

represents the parameterized resulted coordinate gradients on vertex *i*. Then the measure of difference between

$$\frac{\sum_{j\in N_{i}}Area_{j}\nabla_{j}u}{\|\sum_{j\in N_{i}}Area_{j}\nabla_{j}u\|}\;\frac{\sum_{j\in N_{i}}Area_{j}\nabla_{j}\nu }{\|\sum_{j\in N_{i}}Area_{j}\nabla_{j}\nu \|}\;\mathrm{and}\;(d_{i1}\;d_{i2})$$

is expressed as the sum of squared differences. Due to the larger or smaller uncertainty of the principal curvature directions *d*_*i*1_ and *d*_*i*2_ [38, 39], the operator min(•) representing the minimum value is used. Finally $\sum_{i\in V}$ integrating all the vertices on the targeted mesh, metric *E*_v_ is achieved. Metric *E*_v_ is the convex function. Our method, represented by Eq (9), is guaranteed to minimize *E*_v_ and the algorithm would be converged as the iteration deepens. Our algorithm is consistent with [40] in the form of convergence, and requires multiple iterations to converge to a numerical minimum.

$$E_{v}=\sum_{i\in V}\min\left(\begin{array}{l} {\left(\frac{\sum_{j\in N_{i}}Area_{j}\nabla_{j}u}{\|\sum_{j\in N_{i}}Area_{j}\nabla_{j}u\|}-d_{i1}\right)}^{2}+{\left(\frac{\sum_{j\in N_{i}}Area_{j}\nabla_{j}v}{\|\sum Area_{j}\nabla_{j}v\|}-d_{i2}\right)}^{2}\\ {\left(\frac{\sum_{j\in N_{i}}Area_{j}\nabla_{j}u}{\|\sum_{j\in N_{i}}Area_{j}\nabla_{j}u\|}-d_{i2}\right)}^{2}+{\left(\frac{\sum_{j\in N_{i}}Area_{j}\nabla_{j}v}{\|\sum_{j\in N_{i}}Area_{j}\nabla_{j}v\|}-d_{i1}\right)}^{2} \end{array}\right)$$ (9)

## Experiments and discussion

The novel method presented in the paper has been implemented in MATLAB2008a. The experiments were performed on a Windows8 PC with a 2.60 GHz Intel Celeron CPU and 2.0 GB RAM. To compare with our method, several previous boundary-free parameterization methods including classic LABF[18], linear quasi-harmonic parameterization(LQHP)[28], iterative quasi-conformal parameterization (IQCP)[31] and quasi-conformal T-Map(QCTM)[24], have been also realized in experiments on above experimental condition. The surface mesh experimental models include a balls model, a beetle model and an ISIS model.

It is often convenient to express parametric metric distortion based on the singular values *Γ* and *γ* of the 3×2 Jacobian matrix *J*. In parameterization of each triangle *i* on the surface mesh, area distortion and angle distortion could be represented by *Γ*_*i*_*γ*_*i*_ and *Γ*_*i*_/*γ*_*i*_ respectively. We would like to minimize area and angle distortion on all the triangles of the mesh. In our experiments, we represent the total area distortion (angle distortion) of the surface mesh by (*Γγ*)_*m*_ ((*Γ*/*γ*)_*m*_) which is the average value of area distortion (angle distortion) parameterized on all the triangles of the mesh, for reason that the average value is just a determinant of the measurement distortion. Note that: the variance of area (angle) distortion of parameterization on all the triangles of the mesh, represented by (*Γγ*)_*v*_ ((*Γ*/*γ*)_*v*_), explicit the non-uniformity of the distribution of the distortion along the surface geometry and the boundaries (including external and interior boundaries) of the mesh patch; the maximum of area (angle) distortion explicit the most non-uniformity of the distortion in the parameterization. In the paper, to describe the metric distortion of a mesh patch as a whole, we utilized the average value of area distortion (angle distortion) of parameterization to represent the metric distortion, while not considering the variance and the maximum of distortion on all the triangles which only have local significance.

On the other hand, for reason that the relation between the singular values *Γ* and *γ* of the 3×2 Jacobian matrix *J* represents the parameterized metric distortion, we may list the distribution of *Γ* and *γ* on all triangles of a mesh surface in the parameterization, which would reflect fuzzily the metric distortion of a parameterization as a whole. In parameterization, “*Γγ* = 1”represents area-preserving completely, and “*Γ*/*γ* = 1” represents angle-preserving completely. Therefore, utilizing two groups of 3D parameterization data (*Γ*,*γ*,*Γγ*)and (*Γ*,*γ*,*Γ*/*γ*) of all the triangles in the mesh patch, we fitted two 3D surface patches to compare with the plane *Z* = 1(where *Z* represents *Γγ* or *Γ*/*γ*) for reason of more intuitive observation of the authalic and conformal effect during the parameterization. At the same time, the histogram was used to show the overall area distortion and angle distortion.

**Fig 9:**
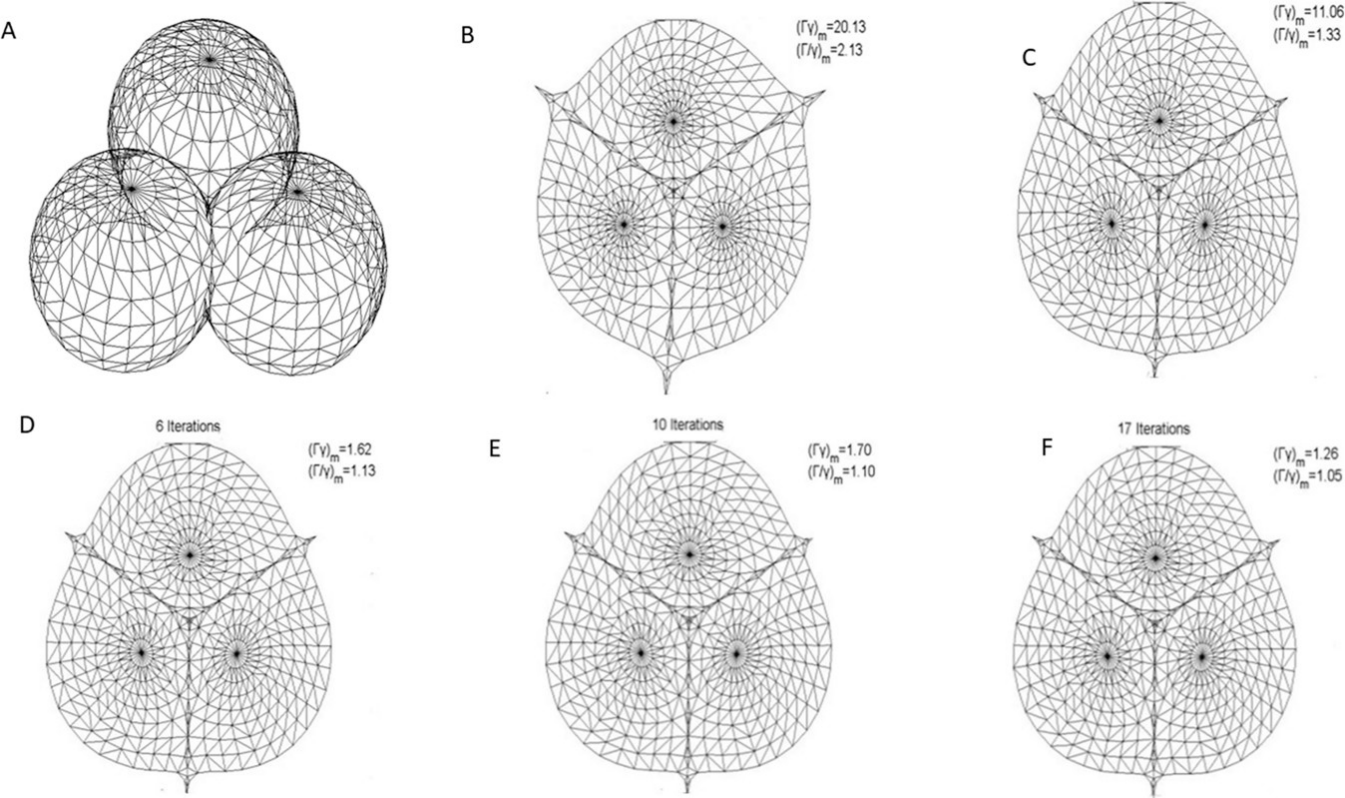
Parameterization experiments on the balls model. (A) original mesh model,(B) result by LABF, (C) result by LQHP, (D) result by IQCP, (E) result by QCTM, (F) result by our method.

One representative kind of model to depict the effect of parameterization is the spherical surface mesh such as the balls model (1032Δ) shown in (Fig 9A). The balls model was segmented along the junction of the three balls surface into three patches each with a disk topology by using the cutting method in [41].

**Fig 10:**
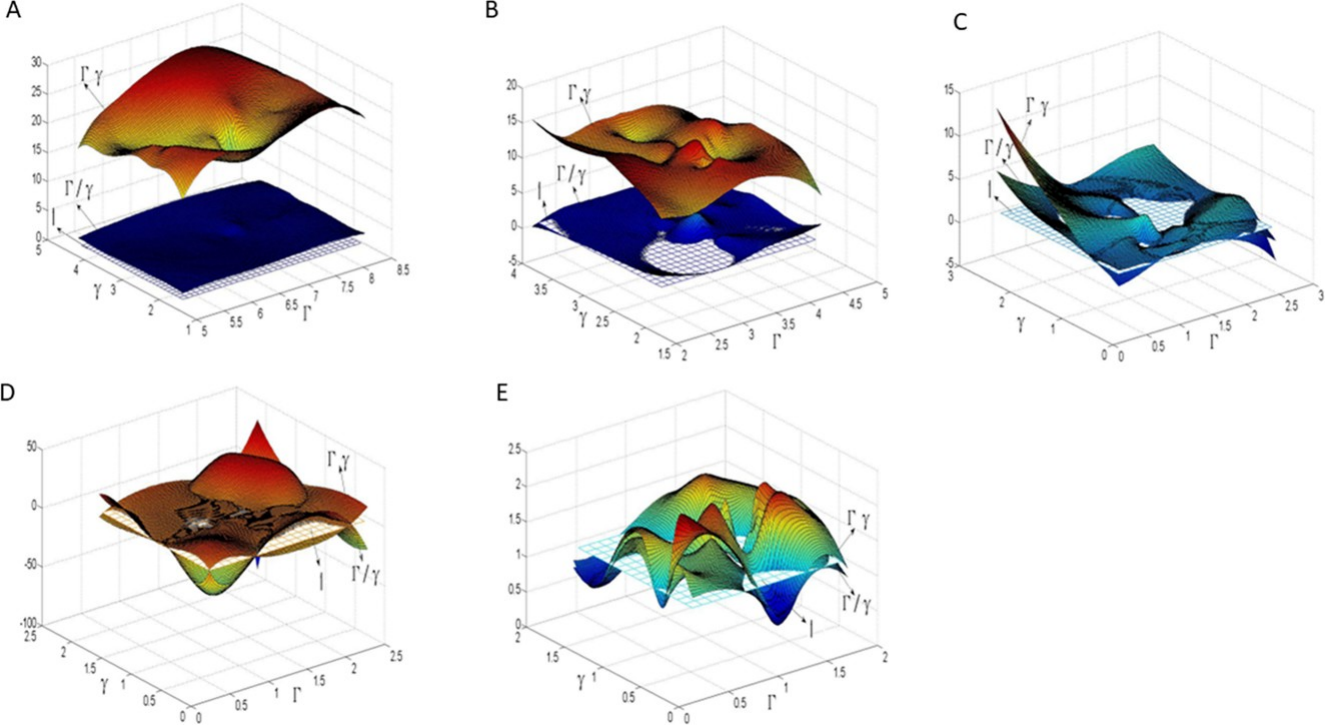
3D fitted surface on the singular values and their relation of Jacobian matrix in parameterization on balls model. (A) by LABF; (B) by LQHP; (C) by IQCP; (D) by QCTM; (E) by our method.

In parameterization, the distribution of *Γ*, *γ* and their relation (i.e., *Γγ* or *Γ*/*γ*) on balls model is illustrated in (Fig 10). By comparing with the plane Z = 1 which represents authalic or conformal parameterization completely, the effect of metric distortion in parameterization can be clearly expressed by fitting the 3D surface of the parameterized data by various methods. As shown in (Fig 10A and 10B), the authalic effects of the parameterizations by LABF and by LQHP are far from satisfying the great distance between the fitted 3D surface and the plane *Γγ* = 1; yet their conformal effects are just passable for its waver along the plane *Γ*/*γ* = 1. Although the 3D fitting surfaces parameterized by IQCP and QCTM wave around the plane *Z* = 1, the rolling vertical amplitude is too large to be acceptable whose changes from 5 to 30 can be seen in (Fig 10C and 10D). The 3D fitting surfaces on *Γγ* and *Γ*/*γ* by our method wave around *Z* = 1 with the amplitude changing from 0.3 to 1.7 as shown in (Fig 10E), which exhibits the best effect among the five methods in authalic and conformal aspects.

**Fig 11:**
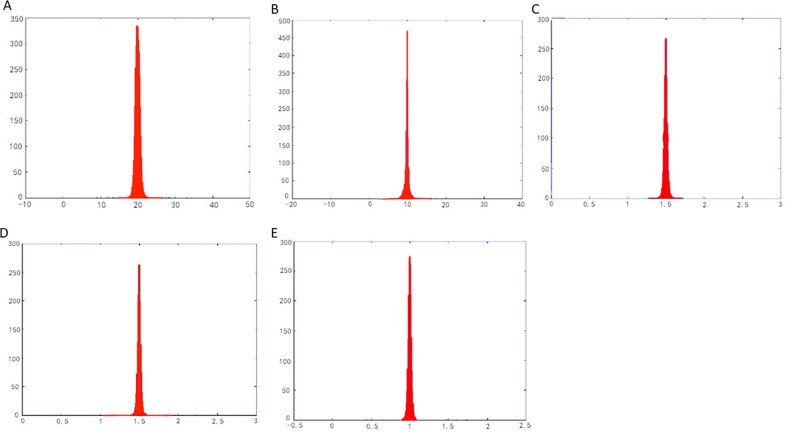
Histograms of area distortions on the balls model. (A) result by LABF, (B) result by LQHP, (C) result by IQCP, (D) result by QCTM, (E) result by our method.

**Fig 12:**
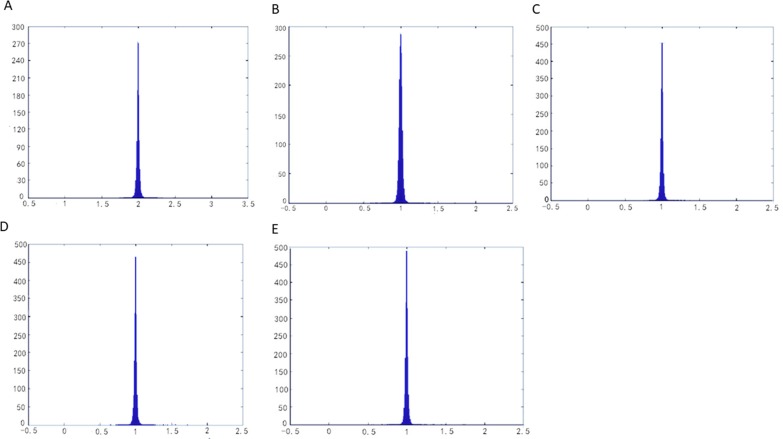
Histograms of angle distortions on the balls model. (A) result by LABF,(B) result by LQHP,(C result by IQCP,(D) result by QCTM,(eE) result by our method.

The parameterization results operated by methods of LABF, LQHP, IQCP, QCTM and ours are displayed respectively in (Fig 9B, 9C, 9D and 9E), from which some features of each method could be found easily by comparison. The average value of area distortion (*Γγ*)_*m*_ of IQCP or QCTM or our method is much less than that of LABF or LQHP: (1.62 or 1.70 or 1.26) vs. (20.13 or 11.06), and that of our method is obviously less than IQCP or QCTM: 1.26 vs. 1.62(or 1.70); in other words, the average value of area distortion of our method decreases respectively 93.7%, 88.6%, 22.2% and 25.9% than that of LABF, LQHP, IQCP and QCTM. Our method also achieves a significantly lower angle distortion (*Γ/γ*)_*m*_ than that of previous methods. The comparison of average value of angle distortion is 1.05 vs.2.13 (or 1.33 or 1.13 or 1.10); in other words, the average value of angle distortion of our method decreases respectively 50.7%, 21.1%, 7.1% and 4.5% than that of LABF, LQHP, IQCP and QCTM. In (Fig 11), the contrasts between the area distortion of the method of LABF, LQHP, IQCP, QCTM and our proposed algorithm show that our proposed algorithm possesses lower overall area distortions. At the same time, according to(Fig 12), our proposed method possesses lower overall angle distortion.

**Fig 13:**
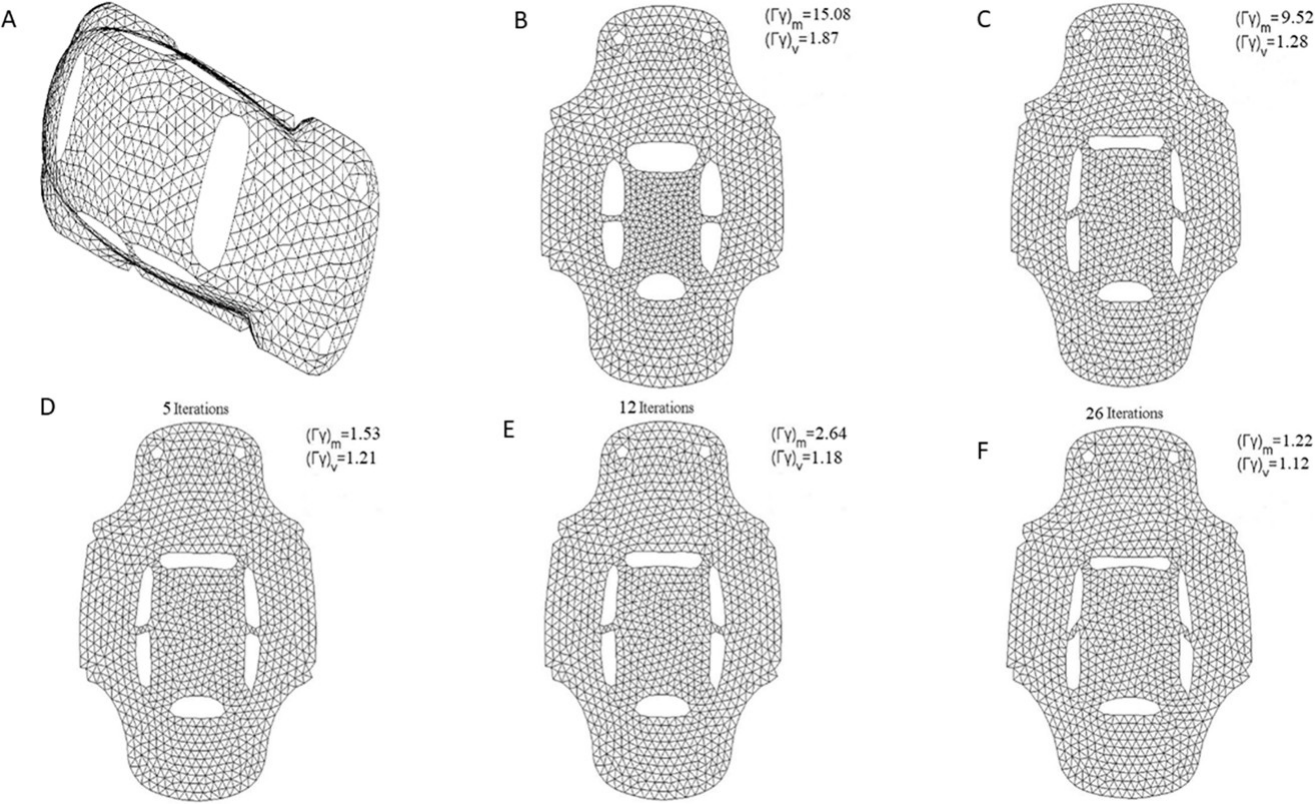
Parameterization experiments on beetle model. (A) original mesh model,(B) result by LABF, (C) result by LQHP,(D) result by IQCP, (E) result by QCTM, (f) result by our method.

Beetle model (1759Δ) (shown in Fig 13A) was also segmented into a few patches with a disk topology by using the cutting method in [41].

**Fig 14:**
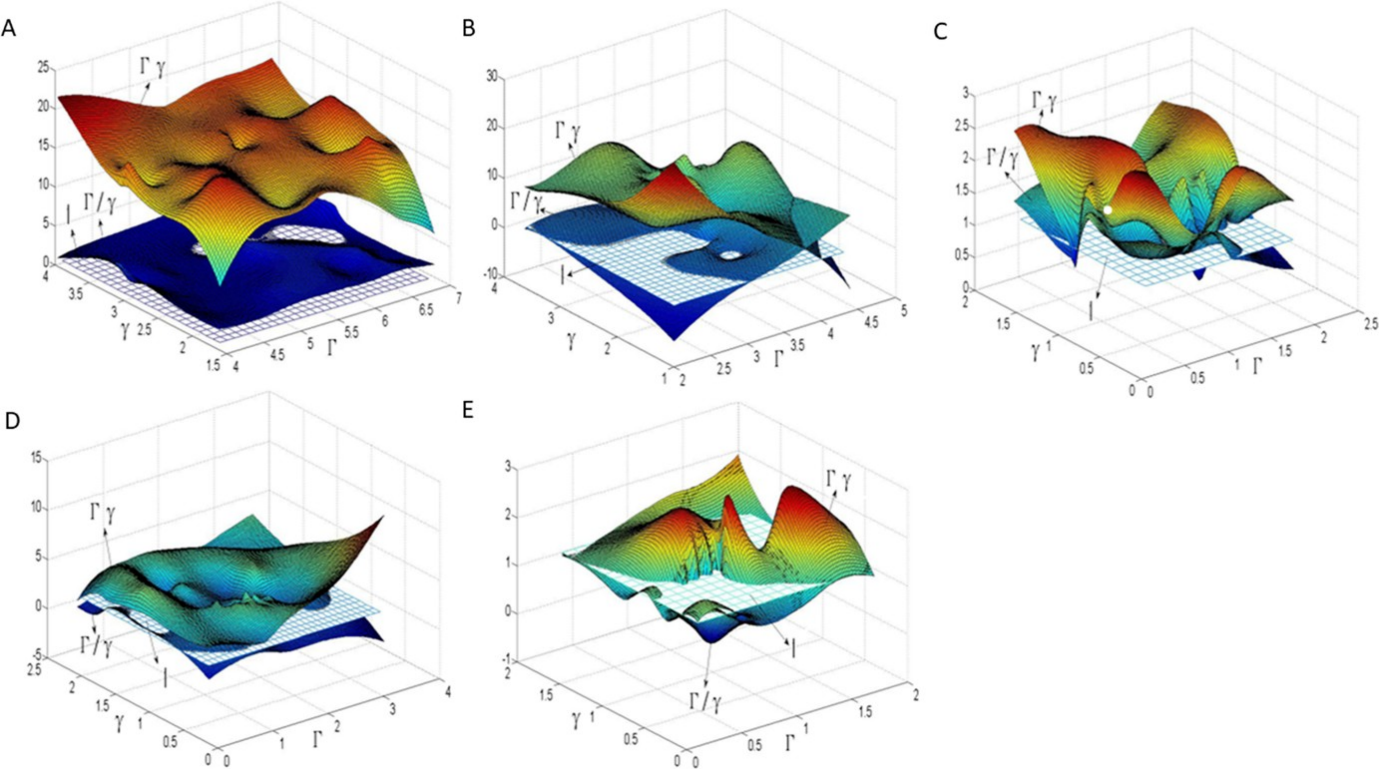
3D fitted surface on the singular values and their relation of Jacobian matrix in parameterization on beetle model. (A) by LABF,(B) by LQHP, (C) by IQCP, (D) by QCTM, (E) by our method.

The distribution of *Γ*, *γ* and their relation on beetle model is illustrated in (Fig 14). Same as balls model, through comparing with plane *Z* = 1, the 3D fitting surface by various methods expresses explicitly the effect of metric distortion in parameterization. Similar to the balls model, the parameterizations of LABF and LQHP is far from authalic effect shown in (Fig 14A and 14B); the waving amplitude is a little or significantly greater on the fitting surface on parameterization data by IQCP and QCTM as in (Fig 14C and 14D). By comparison, The authalic and conformal effects in parameterization by our method on beetle model are the best, whose two fitting surfaces are closed to the plane *Z* = 1.

**Fig 15:**
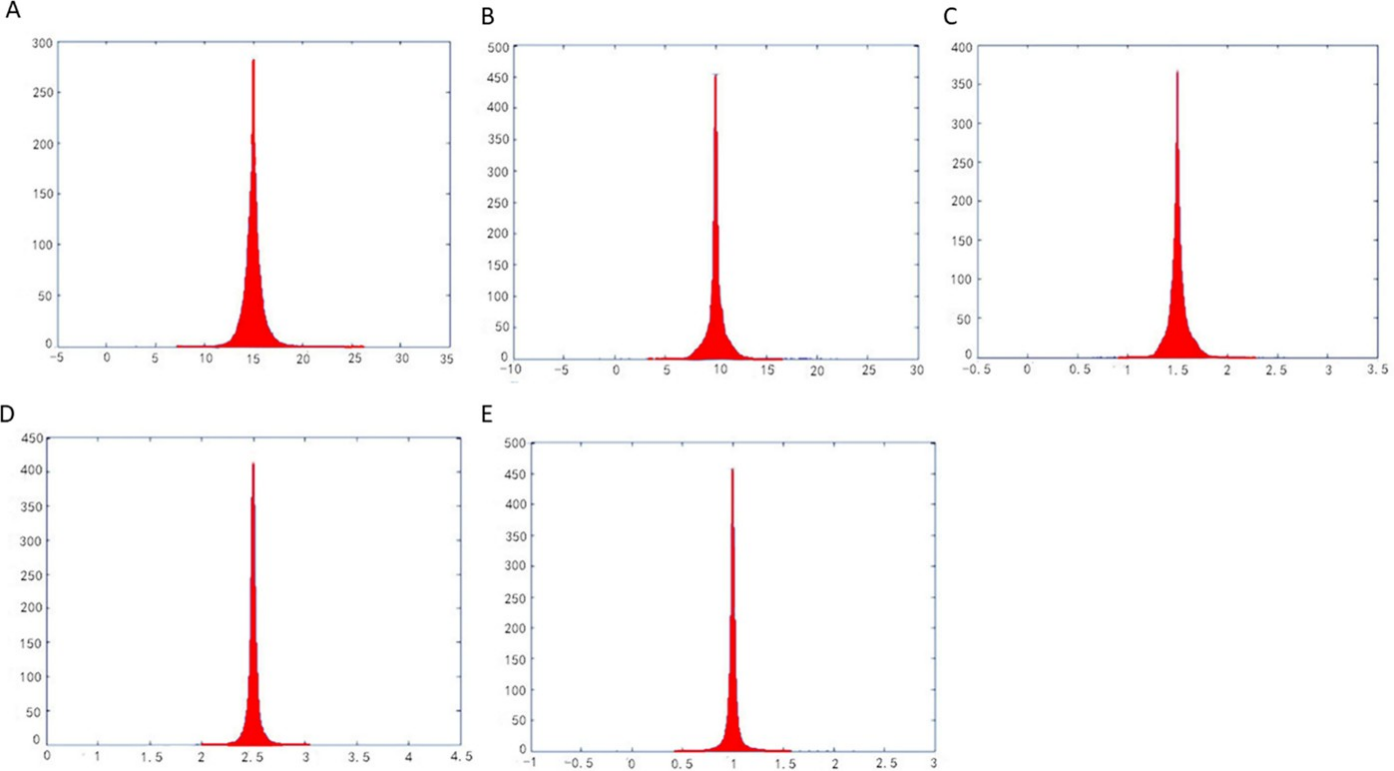
Histograms of area distortions on the beetle model. (A) result by LABF, (B) result by LQHP, (C) result by IQCP,(D) result by QCTM, (E) result by our method.

**Fig 16:**
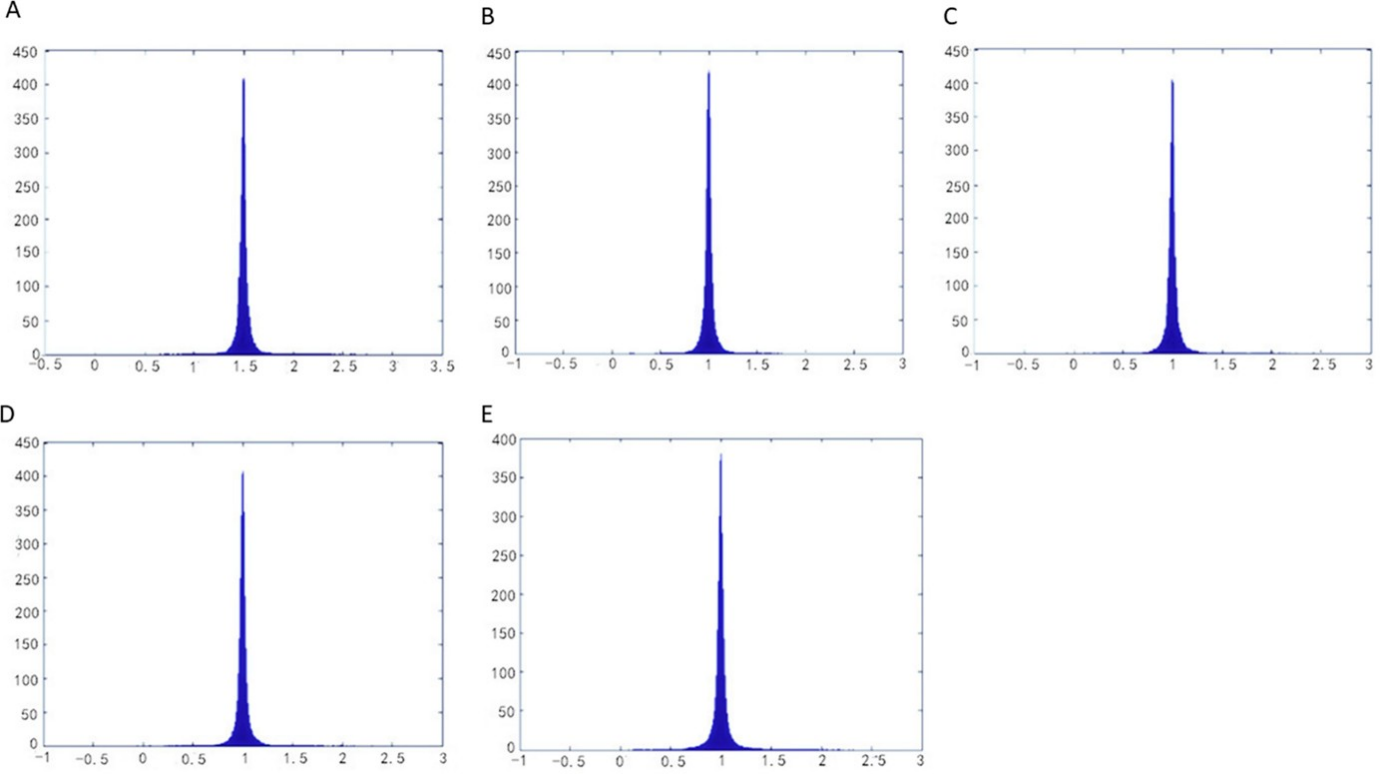
Histograms of angle distortions on the beetle model. (A) result by LABF, (B) result by LQHP, (C) result by IQCP, (D) result by QCTM,(E) result by our method.

The parameterization experiments of Beetle model are illustrated in (Fig 13B, 13C, 13D, 13E and 13F). The average value of area distortion (angle distortion) by our method is lower than that by other four methods (LABF, LQHP, IQCP, QCTM): 1.22 vs. (15.08, 9.52, 1.53, 2.64) (1.12 vs. (1.18 or 1.21 or 1.28 or 1.87)); in other words, the average value of area (angle) distortion of our method decreases respectively 91.9%, 87.2%, 20.3% and 53.8% (5.1%, 7.4%, 12.5% and 40.1%) than that of LABF, LQHP, IQCP and QCTM. According to (Fig 15), compared with the method of LABF, LQHP, IQCP, QCTM, our proposed method also shows lower overall area distortions. At the same time, our proposed method possesses lower overall angle distortions in (Fig 16).

Because the great reduction of our method on average value of metric distortion which is the determining factor to measure the metric distortion. It could be deduced easily from the statistics data on above experiments that the parameterization effect of our method is better than that of other four previous methods. Thus our method is considered to be better than other previous ones.

Taking beetle model as an example, we list various properties of five methods including ours in (Table 1). LABF, LQHP, IQCP, QCTM and ours are all linear. LABF and LQHP are one-time, while IQCP, QCTM and ours are iterative. Because of intrinsic properties, the iterative number of IQCP or QCTM is not very efficient and effective in their later iterative steps. While in our method, multiple iterations could be performed for the following iterative steps to run more efficiently and to obtain more evolutionary results than that of IQCP or QCTM. Thus the parameterized effect in our iterations is enhanced increasingly step by step. Compared with IQCP, QCTM and other two previous one-time methods: 0.75s is much shorter than 0.98s or 2.53s which are running times of other two iterative methods, the parameterization efficiency of our method has been improved significantly; in other words, the running time of our method decreases respectively 23.4% and 70.4% than that of IQCP and QCTM.

**Table 1:** Properties of the methods referring experiments on beetle.

	If iterative	If linear	Iterative termination condition	Iterative number	Running time(s)
LABF	One-time	Yes	−	−	7.34
LQHP	One-time	Yes	−	−	0.25
IQCP	Iterative	Yes	E_IQCP_<20	5	0.98
QCTM	Iterative	Yes	E_QCTM_<12.5	12	2.53
Ours	Iterative	Yes	E_V_<5	26	0.75

**Fig 17:**
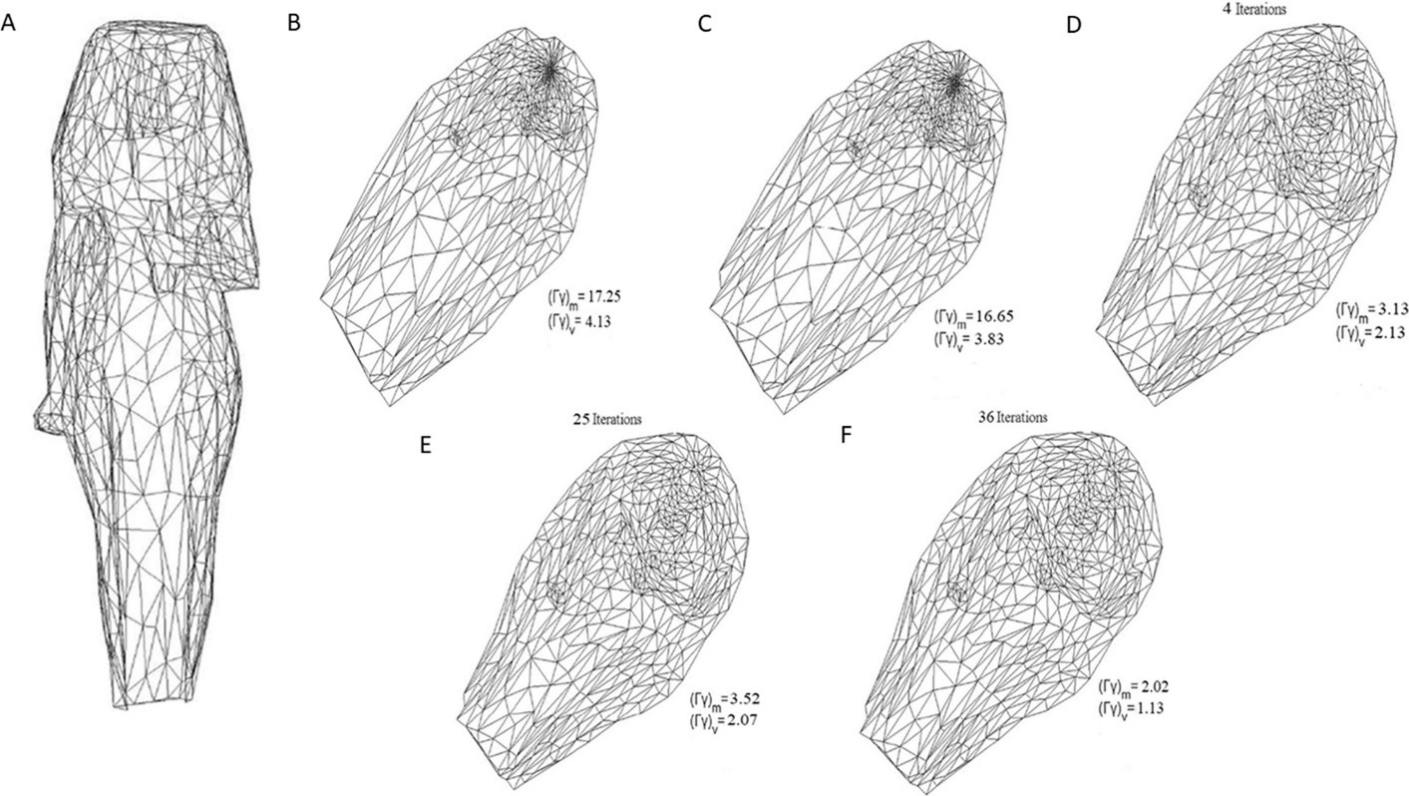
Parameterization experiments on ISIS model. (A) original mesh model, (B) result by LABF, (C) result by LQHP, (D) result by IQCP;,(E) result by QCTM, (F) result by our method.

The above experimental models are simpler 3D surface mesh models. For the more complex 3D surface mesh model, the ISIS model as shown in (Fig 17), the method of this paper also performs very well.

It can be seen from Fig 17 that the average value of the area distortion (*Γ γ*)_*m*_ 2.02 of the method on the ISIS model is less than the average of the area distortion of the IQCP parameterization method by 3.13, and the average value of the angular distortion (*Γ*/*γ*)_*m*_ 1. 13 is also less than the average of the angular distortion of the QCTM parameterization method of 2.07. At the same time, the area distortion and angle distortion are significantly less than the distortion average of the other two methods (is 1/8 to 1/5 of theirs). In addition, it can be seen from (Fig 17B, 17C, 17D, 17E and 17F) that the variance of the area and angular distortion of the method here is also smaller than the distortion variance of the other four methods.

## Limitations

We propose an effective method for boundary-free mesh parameterization. This method has higher efficiency and metric distortion smaller average value for mesh parameterization, which has certain significance in practical applications.

However, the parameterization process usually needs to consider various factors, such as the variance of area (angle) distortion, the maximum of area (angle) distortion and the average value of area distortion (angle distortion). In our method, for reason that the average value is just the determinant to measure metric distortion. we express the total area distortion (angle distortion) of the surface mesh which is the average value of the parameterized area distortion (angle distortion) on all the triangles of the mesh.

Therefore, the experiments in this paper are carried out under the condition that the variance of area (angle) distortion and the maximum of area (angle) distortion are in ideal state. This issue has been demonstrated at the beginning of the "Experiments and Discussions" section. If the influence of them cannot be ignored in the parameterization process. It is necessary to solve the problem caused by this.

## Conclusion

The novel method of boundary-free mesh parameterization is implemented through a series of linear systems. By use of the iterations of quasi-harmonic parameterization on the steps, the angle and area distortion are reduced significantly. The iterations could be converged normally by using new metric, which makes the parameterization results consistent with the geometry of original 3D mesh, to terminate the iterations. Due to its linearity and iterative performance, the method ensures considerable efficiency and good effect. Experiments on several mesh models show that the method is superior to is superior to previous boundary-free parameterization methods. Thus the method can be considered a contribution to the mesh-parameterization field. Furthermore, this research can be extended in some useful ways, for example, the idea of constructing iterations of parameterization might be learned to conduct the global parameterization on an entire closed surface mesh.
